# MicroRNA Nanotherapeutics for Lung Targeting. Insights into Pulmonary Hypertension

**DOI:** 10.3390/ijms21093253

**Published:** 2020-05-04

**Authors:** Susana Carregal-Romero, Lucía Fadón, Edurne Berra, Jesús Ruíz-Cabello

**Affiliations:** 1 Center for Cooperative Research in Biomaterials (CIC biomaGUNE), Basque Research and Technology Alliance (BRTA), Paseo de Miramón 182, 20014 San Sebastián, Spain; scarregal@cicbiomagune.es (S.C.-R.); lfadon@cicbiomagune.es (L.F.); 2 CIBER de Enfermedades Respiratorias (CIBERES), 28029 Madrid, Spain; 3 Center for Cooperative Research in Bioscience (CIC bioGUNE), Buiding 800, Science and Technology Park of Bizkaia, 48160 Derio, Spain; eberra@cicbiogune.es; 4 Ikerbasque, Basque Foundation for Science, 48013 Bilbao, Spain; 5 Departamento de Química en Ciencias Farmacéuticas, Universidad Complutense de Madrid, 28040 Madrid, Spain

**Keywords:** non-coding RNA, microRNA, replacement therapy, antisense-mediated inhibition, mimicRNA, lung targeting, pulmonary disease, pulmonary hypertension, nanoparticle, liposome

## Abstract

In this review, the potential future role of microRNA-based therapies and their specific application in lung diseases is reported with special attention to pulmonary hypertension. Current limitations of these therapies will be pointed out in order to address the challenges that they need to face to reach clinical applications. In this context, the encapsulation of microRNA-based therapies in nanovectors has shown improvements as compared to chemically modified microRNAs toward enhanced stability, efficacy, reduced side effects, and local administration. All these concepts will contextualize in this review the recent achievements and expectations reported for the treatment of pulmonary hypertension.

## 1. Introduction

The first evidence of the regulatory function of microRNA (miRNA) was reported in *C. elegans* by Lee et al. three decades ago [1]. Since this discovery, many examples of these small nucleotide chains were identified in different species, evidencing the existence of a post-transcriptional regulation mediated by RNA molecules and fairly conserved in diverse organisms [2,3]. Numerous laboratories across the world have focused their research in identifying new miRNAs and having a full picture of their biological functions. Interestingly, the public ENCODE project revealed that around 70% of the human genome has biochemical activity, reinforcing the idea that non-coding DNA exceeds protein coding sequences and generating focus on regulatory nucleotides [4]. Similarly to miRNAs, small interfering RNAs (siRNAs) are also noncoding RNAs with an important role in gene regulation [5]. Both miRNAs and siRNAs are short RNA molecules that target messenger RNA (mRNA) and can exert gene silencing. However, siRNAs are very specific with only one mRNA target while miRNAs have multiple targets [5]. Research on siRNA clinical applications has advanced ahead of miRNAs because they are effective in gene silencing and they could involve less potential off-target effects because of this one single target [6]. But, miRNA-based therapies can provide in addition to gene silencing (and elimination of undesired proteins) the restoration of missing proteins to physiological levels. Consequently, these therapies are challenging but also offer more therapeutic opportunities than siRNA-based drugs. For this reason, they are considered today the future of non-coding RNA treatments. In addition, they are currently being applied as quantifiable indicators of pathological states and as molecular biomarkers for early diagnosis. Regular protocols examine differences in the expression of miRNAs as prognostic markers between groups of patients, with and without medical treatments, searching for miRNA targets that can give information about the efficiency and effect of different therapies [7,8]. Regarding the use of miRNAs as biomarker there are already clinical databases, such as the BACAP cohort for pancreatic cancer, that provide the opportunity to identify correlations between the presence/expression of a broad panel of miRNAs. Finally, for some diseases it has been possible to develop miRNA targeted treatments based on these clinical databases. The most advanced treatment in this sense is called miravirsen and it is based on a locked nucleic acid (LNA)-modified oligonucleotide targeting the liver expressed miR-122 for treatment of hepatitis C virus infection [9,10]. 

miRNA-based treatments suffer for low stability and efficiency [11] and they require chemical modifications [12,13] or conjugation/encapsulation in different kinds of nanovectors such as lentivirus, polymeric nanoparticles, or exosomes [14,15,16]. This review intends to highlight the interest of developing more advanced strategies for miRNA treatments; the challenges related with their stability, specificity, side-effects, and interest of their encapsulation within drug delivery carriers.

Specifically, we will illustrate why and how miRNAs could ameliorate the pathologies and complications of respiratory disease, with a special focus being given to the recent achievements in pulmonary hypertension (PH). PH involves a continuous remodeling of the pulmonary vasculature, that is similar to cancer in some aspects [17], leading to muscularization of pulmonary vessels, development of vascular lesions, continuous vasoconstriction, and final heart failure. Current pharmacological therapies only target three pathways that either regulates the vasodilatory/vasoconstriction balance (prostacyclin and endothelin pathway) or the uncontrolled cell proliferation (nitric oxide pathway) [18]. As a result, of these therapies patients can only improve their life quality but not without suffering adverse side effects. This fatal lung disease lacks effective treatments. Therefore, there are compelling reasons to find new molecular targets and novel therapies that reverse the development of the disease. In this context, miRNA-based therapies have shown promising results that we will provide in the following sections while explaining the important role that had played their nanoencapsulation [19].

## 2. Brief Description of miRNA Biogenesis

**Figure 1:**
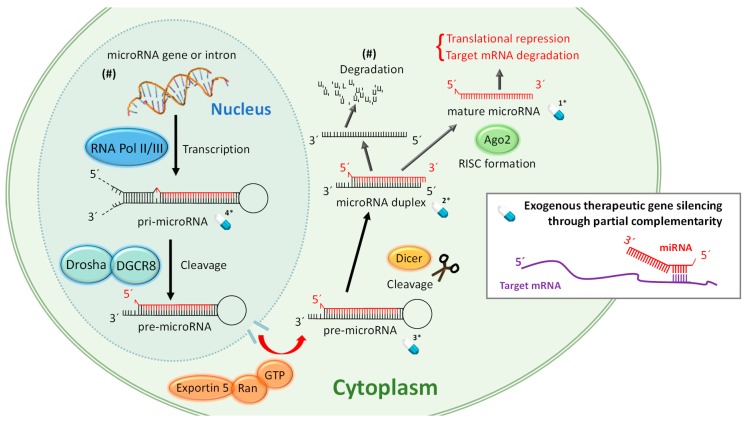
One of the possible mechanisms and pathways of the miRNA biogenesis within eukaryotic cells of mammals. We have marked with a blue pill drawing and a number 1* to 4* the possible miRNA and miRNA precursors that can be targeted for the different therapeutic strategies explained in Section 4. (#) Indicates that this pathway can be different.

To understand the therapeutic strategies based on miRNA, it is important to keep in mind the biological processes required for the biogenesis of mature, functional, and single stranded miRNA shown in Figure 1. Only this mature molecule will be capable of achieving gene silencing through partial complementarity with its target mRNA. miRNA biogenesis involves several biological processes where the correct action of auxiliary factors is crucial but their degree of influence is still under discussion [20]. Discrepancies have been found between species [21], and some authors refer to two pathways [22], but here for the sake of simplicity we have condensed the miRNA maturation process in general terms. Figure 1 attempts to summarize the complex miRNA biogenesis in eukaryotic cells. First, often RNA polymerase III, in the cellular nucleus, transcribes specific DNA sequences, giving rise to long stem-loop structures called pri-miRNA. pri-miRNA has often well conserved terminal loops where auxiliary factors bind to ensure an optimal pri-miRNA processing. One of these factors is hnRNP, an RNA-binding protein that acts by binding the loop of the pri-miRNA to produce a relaxation at the stem [23,24]. Subsequently, Drosha, a RNase-III type enzyme, binds the microprocessor complex DGCR8 and cleaves the nucleic acid to form pre-microRNA, a 60–80 base pairs chain with typical hairpin conformation. After that, the protein Exportin-5 (and probably through alternative modes of transport) is responsible for transporting the pre-miRNA to the cytosol through nuclear pores where it will be further processed until becoming mature and functional [25]. In this compartment, it is cleaved by Dicer, creating microRNA duplexes. These duplex RNAs bind the protein Argonaute (AGO) which is implicated in the separation of the double chain and form the RNA-induced silencing complex (RISC). Finally, the guide strand remains anchored to the complex to fulfil its duty while the passenger strand is decomposed in some cases but it can also stay being functional. Generally, the strand with the less stably paired 5ʹ-end is selected as the mature miRNA. Argonauts are composed of three domains, PAZ, MID, and PIWI. In humans, four isoforms have been found. Recent studies point to the mechanism by which miRNA exerts its action softly differs depending on the binding to the AGO isoform [11].

miRNA-based therapies target at least one of the multiple miRNA biogenesis pathways which gives an idea about the variety of possible therapies. In Figure 1 we have only highlighted miRNAs that can be administered in therapies based on replacement by exogenous nucleic acids and there are already four. If we consider that these four possibilities can be synthesized with different chemical modifications, the options for building novel drugs exponentially increase. In the following sections we will try to give a short overview of the recent and more successful (up to now) therapeutic strategies.

## 3. Key Features of miRNA-Based Medical Applications

**Figure 2:**
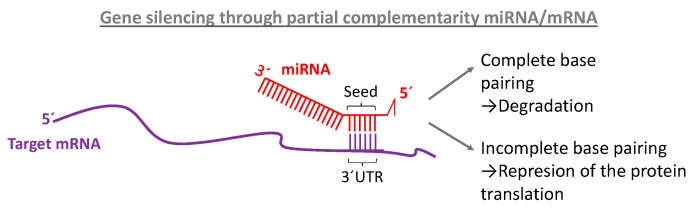
Complementary binding between mRNA and miRNA at the seed region located on the bases 2–8 on the 5´ end of mature miRNA and the 3´UTR of the target mRNA.

miRNAs largely influence homeostasis and their abnormal expression can be related to various diseases although their complete mode of action is not yet understood. In principle, miRNA exerts their function by canonically pairing their cognate mRNA. For the target recognition, a mature miRNA has “seed regions,” short sequences extending from bases 2 to 8 on the tail of the mature miRNA strand on the 5’ end that bind the targeted mRNA as shown in Figure 2. 

These segments match a complementary sequence of the 3’ untranslated region (UTR) of the mRNA, 5’ UTR or within the mRNA coding sequence. The union of the miRNA to the mRNA promotes this oligonucleotide destruction, downregulating the expression of their codified protein. This inhibits the protein synthesis [26]. However, the regulation of the gene expression can be accomplished through different genetic and epigenetic mechanisms. The method of choice varies depending on complementarity. If the miRNA matches perfectly to the target mRNA the interference pathway is activated and the mRNA degraded. But, in the case that the base pairing is incomplete, miRNA hybridize partially to the cognate region of the mRNA, forcing the repression of the protein translation [27]. In addition to these functions, miRNA has been found to be involved in chromatin modification and in the methylation pathway [28,29]. There is a general consensus on the lack of full knowledge about all mechanisms for gene silencing and biological functions of miRNAs and there is a constant release of new information about novel roles of these molecules in human disease [30]. 

Because of the difficulties to establish the precise mode of action of a particular miRNA, mathematical tools have been proposed to discriminate between processes in order to make a practical use of all obtained information toward clinical application [31,32]. In this line, using a theoretical base and computational tools, a system biology approach which combines data-driven modelling and model-driven experiments has been suggested as an efficient method to better understand the regulatory role of miRNAs in coordinating gene expression [33]. This work also underscored the idea that mathematical modelling is necessary for a deep understanding of miRNA’s biological role and highlighted the complexity of the miRNA regulation. This becomes even more important when we take into account that there is a large amount of miRNAs that have not been discovered yet. More than 1 × 10^11^ potential combinations of different miRNAs can be generated with a sequence of only 20 nucleotides. In contrast, only less than 2000 different matured human miRNAs have been identified to date [34]. 

The large amounts of miRNAs holding key biological roles, summed to the coincidences detected between the different species, seems to point out that these molecules are greatly selected and evolutionarily conserved. Thus, the translation of findings from preclinical models is more prospective than other molecular targets. miRNAs serve as biomarkers of disease progression and are targets for therapeutic intervention [26]. Regular protocols for biomarkers selection examine the differences in the expression of these molecules between groups of individuals in search for syndrome targets and evaluation of the effect of therapies.

Some of the investigated disorders are: heart failure [35], pancreatic cancer [36], hepatitis B [37], or asthma [38]. Examples of therapies that have been examined using miRNAs as biomarkers are: Vandetanib for advanced medullary thyroid cancer [39], oxaliplatin and capecitabine chemotherapy for advanced gastric [40], tofacitinib treatment for rheumatoid arthritis [41], pegylated interferon therapy for hepatitis B [10], and adalimumab for early rheumatoid arthritis [42]. 

## 4. Types of miRNA-Based Therapies

The association of siRNAs and miRNAs with many diseases and their implication in modulating key aspects of gene silencing has brought these two molecules into the forefront of new therapeutic interventions [27,43]. More than 20 clinical trials have been started in the last decade applying siRNA- and miRNA-based drugs for new therapeutics [44]. The first siRNA medication, patisiran (commercialized as Onpattro in the USA), has been approved in 2018 by the FDA. miRNA-based therapies are less advanced but some are already in clinical trials such as miravirsen which is an inhibitor or miR-122 and many more that are in the preclinical stage [44]. Although the modes of action of siRNAs and miRNAs are different both oligonucleotides are single stranded and form RISC complexes. Therefore, some of the strategies developed for an efficient siRNA drug delivery are being used to push the development of miRNA-based therapies.

All miRNA-based therapies present relevant advantages compared to other drug classes. First, they act fast and can alter gene expression of multiple transcripts at once [44]. Moreover, the fact that these are endogenous compounds decreases their reactivity and immunogenic responses. miRNA and their targets are conserved and highly specific, diminishing adverse effects. Finally, miRNA can specifically suppress the translation of any gene, including intracellular targets. They can modulate drug targets that are inaccessible by conventional small molecule inhibitors and antibody based-therapies [45].

However, the expected clinical success was not achieved until recently after the major hurdles of these therapies were overcome. These major issues included: (a) Inefficient binding affinity for complementary sequences, (b) inefficient pharmacokinetics and sub-optimal biodistribution, (c) low cell membrane penetration, and (d) enzymatic degradation by nucleases in vivo [46]. Many miRNA-based therapies are synthetic oligonucleotides, chemically modified to enhance their stability, tissue targeting, uptake efficacy and binding affinity [46,47]. In addition, they are encoded as prodrugs, in order to protect the active substance from the degradation mechanisms of exogenous genetic material that exist in eukaryotic cells [48]. 

The most common modifications within the miRNA chain are led first to resist degradation by RNA nucleases and slow their removal in vivo by the liver. These chemical modifications alter the internucleotide connections or replace backbone phosphodiester for phosphorothioate, boranophosphate or peptide bonds. miRNAs can be also modified to improve cell and tissue targeting by decorating miRNAs with receptor-mediated endocytosis pathways. On the other hand, cell uptake can be improved by incorporating, at the 3’ end, lipids or cholesterol which increases the permeability through the cellular lipid bilayer. Chemical functionalization with pH sensitive motifs have also been incorporated to synthetic miRNAs, typically internalized by cells in endosomal compartments, to escape and reach the cytosol where they exert gene silencing. The strategies to chemically modify oligonucleotides have been the subject of many recent reviews and debates about their influence on the pharmacological properties or miRNA-based therapies since often they reduce their efficiency [46,47].

In 1997, the first chemical approximations of modified RNAs were reported by Takesi et al. They designed LNAs that are RNA analogues where the ribose residues are modified with an extra bridge that connects oxygen 2′ with carbon 4′ and paired specifically with their target miRNA [49,50]. In 2005, Krutzfeldt et al. named a novel class of chemically engineered oligonucleotides “Antagomirs.” These molecules were described as engineered siRNA with certain drug-like properties complementary to miRNA. They described a method to modify chemically single-stranded RNA analogues conjugated to cholesterol that was able to bind specific miRNA to achieve therapeutic silencing of an endogenous gene in vivo [51]. A couple of years later, Wang et al. developed the miRNA mimic technology (miR-Mimic). With this approach is possible to generate double-stranded miRNA-like RNA fragments that can bind specifically to its target mRNA and produce posttranscriptional repression of the gene, mimicking an endogenous miRNA but acting as a gene-specific target [52,53].

Because of the existence of different chemical strategies to restore the physiological levels of miRNAs it has been possible to establish three different kinds of miRNA-based therapies (that can be administered within nanoparticles or bare):

(a) Replacement therapies

In this case, the non-natural miRNA is the drug. Exogenous miRNAs are delivered into the organism to substitute underexpressed miRNAs (Figure 1). Theoretically, an artificial single stranded and mature miRNA could be used to replace the downregulated endogenous miRNA but this strategy has shown inefficient results probably because of the low stability of the molecule. Full length pri-mRNA has also been assayed as possible therapy but it was inefficient because pri-mRNA requires processing at the nucleus. Typically, the mimic miRNAs used in replacement therapies are duplex miRNAs or pre-miRNAs. Viral vectors are other kind of replacement strategies and probably the most efficient nanotherapies in transfecting cells though their application raises some safety concerns. 

(b) Antisense-mediated inhibition therapies

Herein, the drug is an inhibitor called anti-miRNA of a specific and endogenous miRNA. These therapies intent to inhibit overexpressed miRNAs involved in pathogenic states of target diseases. The already mentioned LNAs or Antagomirs are examples of inhibition therapies. Miravirsen, the first LNA-based drug in clinical trials, antagonizes microRNA-122 which is a therapeutic target for the chronic hepatitis C virus infection [54].

(c) Therapies based on influencing the auxiliary factors of miRNA biogenesis

These therapies, less popular than the two aforementioned, target the auxiliary factors of miRNA biogenesis such as the AGO proteins [55]. Schmidt et al. developed miRNA-specific AGO2 protein inhibitors. They act both by antagonism to the active site of the enzyme and by blocking the short recognition sequence to the solvent-exposed target microRNAs seed region [56].

## 5. Administration of miRNA-Based Therapies and the Relevant Contribution of Nanotechnology

**Figure 3:**
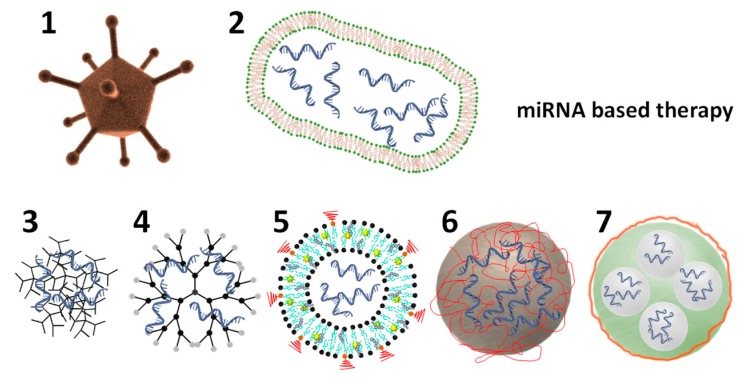
Schematic representation of different nanovectors loaded with miRNA-based therapies. (**1** & **2**) depict an adenovirus and an EDV^TM^ nanocell. (**3**) Depicts a nanovector made of a positively charge polymer and negatively charged miRNA-based therapy. (**4**) Depicts a dendrimer. (**5**) Depicts a multifunctional liposome, loaded in its interior with miRNAs and functionalized with PEG (in red) on its surface and a contrast agent (yellow star) within the lipidic bilayer. (**6**) Depicts a solid nanoparticle coated with a positively charged polymer (in red) and the miRNA-based therapy via self-assembly. (**7**) Depicts a porous solid particle which encapsulates the miRNA-based therapy within its pores and it is coated to seal the pores. Therapeutic miRNA is depicted in blue and polymeric coatings in red.

miRNA-based therapies are administered using diverse approaches. The simplest strategies are the local, systemic, or targeted administration of chemically modified miRNAs or anti-miRNAs [57,58]. Despite considerable progress has been achieved with these three administration methods (i.e., enhanced bioactivity and decreased side effects) there are several issues to be addressed for a more extended clinical translation. One of them is the deficient ability of AGO proteins to recognize artificial miRNAs for the efficient formation of the silencing complex RISC. This is often due to the excessive chemical modifications incorporated into miRNA mimics. On the other hand, anti-miRNA-based therapies usually require a high dose in vivo which induces higher risk of negative side effects. Nevertheless, the most critical obstacle to be overcome is the inefficient biodistribution in vivo regarding cell uptake and organ-specific delivery. Thus, there is a great requirement to develop safe and efficient nanovectors for miRNA-based therapies and in particular for treating PH [59,60]. Figure 3 shows the schematic representation of some of the already used nanovectors for delivery of miRNA-based therapies.

### 5.1. Biological Delivery Nanovectors

#### 5.1.1. Virotherapy

Virus-like nanovectors (e.g., adenovirus, lentivirus, etc.,) are extensively used for delivering miRNAs because they dispense miRNAs more efficiently than other carriers [61,62,63]. They also permit the transduction of multiple miRNAs and their physicochemical properties can be partially modified by incorporating moieties such as the antifouling polymer polyethylene glycol (PEG) [64]. 

However, it has been reported that they can induce mutagenic effects causing cancer and undesired immune responses which limits their clinical application, so their real translation into clinics is controvertible [65].

#### 5.1.2. Extracellular Vesicles and Nanocells

In contrast, extracellular vesicles (EVs), considered as drug delivery carriers, are in ongoing clinical trials aiming to translate them from basic research to clinics. The main advantage of these nanovectors is that they can be obtained from patients avoiding toxicity [66]. They are lipidic bilayer vesicles naturally released by cells which size varies from 10 to 10000 nm. EVs are often polydispersed in size and can be further functionalized with nanoparticles, for example for therapy [67]. They are currently being used as successful drug delivery systems for miRNAs into particular cells [14,16], such as hepatocytes or macrophages [68]. Interestingly, they have been recently applied to demonstrate the therapeutic effect of miR-181a-5p/miR-324-5p against PH in vivo [19]. They are supposed to have diminished side effects as compared to chemically engineered carriers because of their controllable origin. However, their exact composition is uncontrolled. They contain active molecules with unknown effects for the organism. Their properties are dependent upon their cellular origin and separation conditions which complicates the establishment of general protocols for their extraction, mass production, and characterization toward ensuring reproducibility and safety. These are the main challenges of this promising technology for miRNA delivery [69]. Other very promising biological carriers for miRNAs (in clinical trials) are the bacterially derived EDV^TM^ nanocells (scheme 2 in Figure 3). In cancer, they have been able to carry and deliver efficiently miRNAs to tumors and stimulate the adaptive immune system augmenting their antitumoral response [8]. These carriers are also biological lipidic bilayers with unknown effects on the body.

### 5.2. Engineered Synthetic Nanovectors

Synthetic nanovectors (schemes 3 to 7 in Figure 3) can be finely designed in the nanoscale and manufactured in a large scale owing to the advances achieved in nanotechnology in the past decades [70]. Encapsulation of miRNAs has been mainly carried out using polymers and lipids [71,72,73]. But solid particles such as calcium phosphate have been used as efficient carriers. Often the driving force for the entrapment of negatively charged miRNAs is the electrostatic attraction with cationic lipids or polymers. Physical caging is also another efficient way to entrap miRNAs in empty cavities. 

#### 5.2.1. Lipidic Nanotherapeutics

Lipofectamine is considered the “gold standard” for nucleic acid transfection. It is a lipid-based nanovector composed of positively charged cationic lipids. Often it is called a “liposome,” however it is unclear whether it forms a hollow shell vesicular structure. It has been used extensively to transfect miRNAs and is one of the examples of nanovectors already used to treat PH and other pulmonary diseases. Its high transfection efficiency has been explained by Cardarelli et al. who claimed that these nanoparticles can prevent lysosomal degradation because of their atypical Brownian motion within cells [74]. Other similar commercially available cationic lipid-based vectors are Invivofectamine, NeoFX, or Oligofectamine.

Liposomes are the most advanced and sophisticated delivery systems for miRNA delivery. They are multilamellar or unilamellar spherical vesicles made of phospholipids composed of a hydrophilic head group and a hydrophobic tail. Depending on the chemical nature of the main hydrophilic group, we can differentiate between positive, negative, and neutral charged liposomes. The first generation of liposomes for gene delivery, able to successfully transfect cells, were composed by phospholipids with a positively charged head group [75]. The encapsulation efficiency is enhanced for liposomes with positive charge as compared with negative or neutral liposomes because of the electrostatic attraction with the negatively charged miRNAs. However, the use of permanently charged cationic lipids can induce cellular and inflammatory toxicity, fast plasma clearance, aggregation, and accumulation in lungs, liver, and spleen. The stability, transfection efficiency, and cellular toxicity is determined in a large extend on the lipid composition, so many novel formulations of lipids and liposomes have been developed in the past decades. Ionizable cationic lipids have been chemically designed to produce pH sensitive liposomes able to complex the negatively charged miRNAs at low pH. Interestingly, they shift their charge to neutral charge at physiological pH toward reduced aggregation, toxicity, and electrostatic adherence to cellular membranes. In addition, this charge reversibility triggers a pH-induced release within the acidic endosomal compartments, hypothetically by destabilization of the endosomal membrane because of the electrostatic interaction between the positively charged lipids of liposomes and the negatively charged lipids of the endosomal membrane. Liposomal miR-34a mimics, based on this kind of amphoteric lipids, have successfully entered clinical trials to treat patients with advanced solid tumors [76]. The last advances in liposomal RNA delivery have been assertively reviewed by Peer et al. [77].

#### 5.2.2. Polymeric Nanotherapeutics

Using polymers as the building blocks of nanotherapeutics has the advantage of tailoring its sustained and controlled drug delivery properties [78]. Examples of polymers that form complexes with nucleic acids through electrostatic interactions are poly(ethylenimine) or poly(allylamine) [79]. Recently dendrimers, which are highly symmetrical hyperbranched polymers, have also been applied as drug delivery systems for miRNAs. In this case, the encapsulation is due to both hydrophilic and hydrophobic interactions [80,81]. Solid polymeric nanoparticles such as the FDA and EMA-approved poly(lactic-co-glycolic acid) (PLGA) are widely used for sustained drug delivery of hydrophobic drugs encapsulated in the interior. However, they can also entrap inside hydrophilic molecules such as siRNAs or miRNAs applying a double emulsion synthetic method or adsorb them on their surface by electrostatic interactions [82,83]. Many different types of polymeric particles can be applied to entrap and release miRNAs but have not yet been assayed. For example, nanogels are good candidates that have demonstrated decent delivery efficiency for siRNA transfection and would work similarly for miRNA [84]. These nanovectors are crosslinked polymeric networks that have high water content and large mesh size. Its advantage over other possible miRNA carriers is its mild conditions for encapsulation of labile drugs and their stimuli-responsive properties [85].

#### 5.2.3. Inorganic Particles as Nanotherapeutics

Inorganic silica and calcium phosphate particles are also promising carriers for miRNA delivery. Both are innocuous to the body and are degraded by hydrolysis, delivering their content. Silica nanoparticles are known for the easy modification of surface chemistry, but also for the variety of sizes, shapes, and structures that can be built with this material. Especially interesting are the porous particles and hollow shells that can host and deliver miRNAs from their hollow cavities [86,87]. Calcium phosphates are among the most widely used pharmaceutical excipients and there are notable examples of miRNAs delivery from this type of particles [88]. They have been even functionalized with targeting moieties for in vivo cell targeting. miRNAs are entrapped in these particles by simple coprecipitation or diffusion under mild conditions [89]. However, they have not yet been applied to pulmonary hypertension. 

In this review, we only give a brief summary of the promising nanotherapeutics that could be applied for the encapsulation and delivery of miRNA-based therapies. But often advanced delivery systems are composites. The stealth properties to escape the reticuloendothelial system are obtained mainly by polymer coating (e.g., polyethylene glycol). Targeting can be accomplished by decorating the nanoparticle surface with antibodies, peptides, and other small molecules. And multifunctionality is often sought by combining metallic nanoparticles with other materials such as Nanoplex developed by Wu et al. This miRNA nanovector composed of pH sensitive micelles and superparamagnetic nanoparticles for magnetic resonance imaging was used for combination therapy with two different mimics (miRNA-29b and miRNA-122) against hepatic fibrosis [90]. In conclusion, there is a broad variety of possible drug carriers but it must be assumed that every nanovector has its pros and cons. In this regard, studies such the developed by Osorio et al. comparing the loading, release and therapeutic effect of three different miRNA nanotherapeutics (liposomes, PLGA, and EVs) are of special interest in the field [91]. 

Although the application of nanotherapies to treat PH is in an early stage, due to the dysfunctional and hyperpermeable vascular endothelium, they could represent a powerful tool to bringing PH therapies, such as miRNAs, to novel cellular targets, as has been assertively reviewed by Segura-Ibarra et al. [60].

## 6. Administration Routes for Lung Targeting

The advantage of targeting lung diseases with miRNA-based therapies as compared with other organs is that it is possible to directly administer these compounds through the respiratory track which limits side and off-target effects [92]. In this context, Scholosser et al. have recently demonstrated that direct administration of mimic miRNA therapeutics via the oronasal or the trachea depositions (with or without aerosolization) are efficient methods for direct targeting of the lungs [58]. In this study, they compared five different direct administration strategies (intratracheal liquid instillation, intratracheal aerosolization with and without ventilator assistance, intranasal liquid instillation, and intranasal aerosolization) with three different systemic strategies used as controls (intravenous, intraperitoneal, and subcutaneous delivery) and demonstrated that all lung-targeted strategies showed lung-selective miRNA mimics uptake as compared to systemic administered mimics. Moreover, they showed that intratracheal administration of a liquid miRNA formulation provided the highest lung specific delivery up to 4 orders of magnitude more than the control strategies. This work highlights the impact of the administration method on the therapeutic outcome and the cost-effectiveness of miRNA mimics. 

A second aspect and less advantageous than direct deposition to take into consideration for lung-specific delivery of miRNAs is their elimination by intrinsic pulmonary clearance mechanisms, including enzymatic degradation, quick absorption into the blood, and ingestion by the alveolar macrophages. In this context, nanovector-based delivery systems such as lipid or polymer nanoparticles are used to minimize pulmonary clearance mechanisms, enhance the therapeutic effect, and achieve specific cell targeting [76]. Although cell targeting remains a considerable challenge in nanomedicine, novel and more specialized delivery technologies are continuously being developed, so miRNA technology would benefit from it [93]. 

## 7. miRNA-Based Therapies in Pulmonary Diseases

Altered expression of miRNAs has been reported for many lung diseases such as asthma, pulmonary fibrosis, and lung cancer [94]. To the best of our knowledge there are no miRNA-based therapies for lung diseases currently accepted by the regulatory agencies, although the same company with patisiran in the market (Ainylam Pharmaceuticals) has finished a phase IIb clinical trial with a siRNA aerosolized therapeutic solution for the treatment of respiratory syncytial virus infection during lung transplantation. Despite this, the promising results obtained in animal models treated with miRNA based therapies have brought great hope in the field [95]. Table 1 summarizes few of reported miRNA-based therapies for lung diseases emphasizing on the characteristics of the treatment such as the type of treatment, the use of nanovector, the phenotypic effect, and the application.

## 8. miRNA-Based Therapies in Pulmonary Hypertension

**Figure 4:**
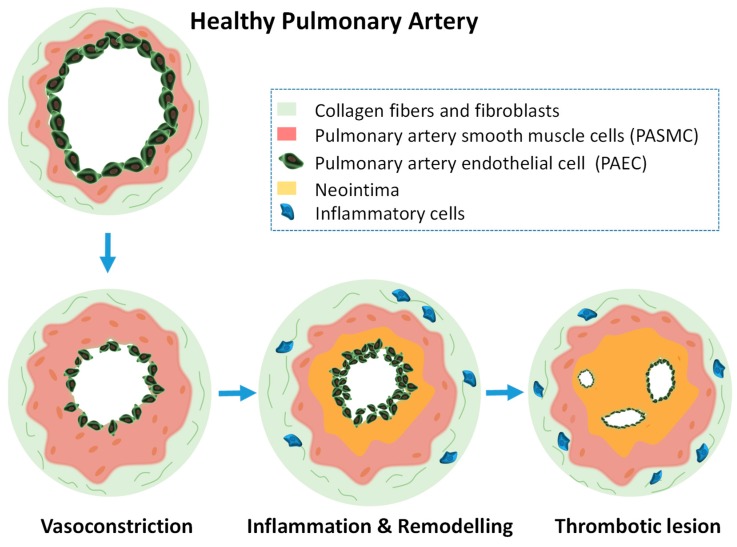
Schematic drawing of a transversal section of a pulmonary artery developing PH and the structural alterations in the different cellular levels. Initially smooth muscle cells excessively proliferate producing vasoconstriction. After that, inflammation, remodeling, and thrombotic lesions will follow.

Pulmonary hypertension (PH) is a condition characterized by increased mean pulmonary arterial pressure. During the compensatory phase, the right ventricle of the heart deals with pressure overload to maintain the blood flow by chamber transformation. This translates into an adaptive increase in artery wall thickness which involves an uncontrolled cell proliferation. Molecular mechanisms underlying the physiopathology have not been completely elucidated but since there is no cure for this fatal disease it urges to find methodologies for the selective detection and treatment of PH [100]. PH has been classified in five different groups which depend on the origin of the disease. Pulmonary arterial hypertension (PAH) is the commonly the most studied case of PH. In PAH, the small arteries (and in some cases also the venules) in the lung are obstructed due to different reasons such as HIV infection, autoimmune disorders, and others but it can be also idiopathic. All PH groups courses with endothelium dysfunction and vascular remodeling in the lungs which has been extensively described that is accompanied by a metabolic reprogramming in the heart and the lung only explored until recently [101,102,103]. It is known that arterioles can undergo vasoconstriction imbalance, cellular remodeling, thrombosis, and cell proliferation. Pulmonary artery endothelial cells (PAECs), pulmonary artery smooth muscle cells (PASMCs), myofibroblast, pericytes, platelets, and inflammatory cells play key roles during the disease development. The vascular remodeling during PH involves PAECs dysfunction, such as proliferation, interaction with PASMCs, and transdifferentiation and the accumulation of PASMCs in obstructive vascular lesions [104,105]. Figure 4 shows schematically a transversal section of a pulmonary artery developing PH.

Currently, PH treatments only help to relieve the symptomatology and do not stop the progression of the disease. The medical therapies available for the treatment of PAH include calcium channel blockers, endothelin receptor antagonists, phosphodiesterase type 5 inhibitors and soluble guanylate cyclase stimulators, prostacyclin analogues, and prostacyclin receptor agonists [106]. miRNA dysregulation has been correlated to the physiopathology of PH and the use of miRNA-based therapies has emerged as new hope toward the reversion of PH symptomatology [107]. Table 2 summarizes a list of reported miRNA that could be efficient therapeutic targets for the treatment of PH. In many aspects, PH disease development resembles tumorigenesis. In PH and cancer the hyperproliferative and apoptosis resistant phenotype is partially related to impaired mitochondrial dynamics and in both cases there is a metabolic dysfunction (Warburg effect) characterized by a shift from oxidative glucose metabolism to aerobic glycolysis which provides the survival conditions for highly proliferating cells [17]. Given these similarities multiple miRNAs that are aberrantly expressed in cancer are also dysregulated in PH. For instance, miR-204 and miR-205 are downregulated in pulmonary artery small muscle cells (PASMs) in PAH as well as in many cancers [108]. In both cases the downregulation of these miRNAs induces metabolic dysfunction and enhanced PASMCs proliferation. MiR-204 and miR-205-based treatments have demonstrated to be efficient for decreasing cell proliferation and metastasis in few cancers and recently it has been reported their potential use for reversing PAH in a murine model [109,110]. Its encapsulation and targeted release in the lungs could be advantageous for preventing the miRNAs being filtered by the kidney, decrease off-targeted effects, and improve intracellular delivery.

Table 2 shows a small portion of the possible miRNA targets and some of their reported targeted genes that could be used to treat PH. Considering that only for dysfunctional PAECs more than 20 different dysregulated miRNAs have been already reported, it will become essential to use in silico approaches to identify the most relevant miRNA targets for developing novel treatments against PAH [126]. In this context, we referred readers to the work recently published by Bonnet et al. [95].

Currently, the treatment of PH with miRNA-based therapies is still in an early phase, similar to siRNA-based therapies [127]. There are no clinical trials going on yet, but fortunately all reported pre-clinical trials up to now have shown significant beneficial effects and some of them have demonstrated an evident decrease of the arteriopathy and cardiac dysfunction. We have collected these examples in Table 3.

As can be seen in Table 3, some studies aimed at determining the possible therapeutic effect of a target miRNA using bare miRNA mimics or Antagomirs as a first assay. This was the case of the study carried out by Pullamsetti et al. in 2012. However, most of the reported research has been done using nanovectors known to improve transfection efficiency such as commercial lipofectamine or viral vectors [110,130]. For instance, in the study of the potential use of miR-145 as a therapeutic target, Caruso et al. was first evidencing the upregulation of miR-145 in humans with PAH and in animal models of PAH. Then, they used antimiR-145 delivered with commercial lipofectamine and showed that it prevented arteriopathy in hypoxic mice. Owing to this study, some years later McLendon et al. reported effective delivery and retention in the lungs of antimiR-145 loaded in more sophisticated cationic liposome-based nanovectors made of polymer-functionalized lipopolyamines that provided stealth properties [7]. Importantly, their results showed an evident decrease of right ventricle pressure, decreased artery muscularization, and lower degree of cardiac dysfunction in murine models of severe PAH while avoiding off-target effects and toxicity. These two last features are essential for the translation of miRNA therapies into clinics but they are still a challenge in the field.

The dose of miRNA required to obtain a therapeutic effect depends on the animal model of PH and many other factors. However, we could draw some general conclusions from Table 3. Comparing two different miRNA therapies in mice housed in hypoxic conditions, reduction of arteriopathy could be achieved in both cases after 2–3 intravenous injections per week for 2 weeks (6 injections in total) [19,128]. When miRNAs were not encapsulated, a higher concentration in treatment, 8 mg/Kg as compared with 2 mg/Kg, was required for the administration of Invivofectamine. In rat models with monocrotaline-induced PH or hypoxia and Sugen, the therapeutic effect was achieved only after 2–3 intravenous injections. Similarly, the concentration of miRNA from the injections was lower for encapsulated therapies (2 mg/Kg vs. 5 mg/Kg) [7,128]. The dose could also decrease with direct administration to the lungs, as demonstrated by Courboulin et al. [109]. Only two nebulizations (1 per week) of encapsulated miRNA mimics were required to obtain a therapeutic outcome of miR-204a mimics in a PH model with monocrotaline. This administration method could also avoid some of the potential off-target effects of miRNA-based therapies, but this has not yet been confirmed. Despite the lungs offer the advantage of local delivery of therapies, as can be seen in Table 3, the majority of the miRNA treatments are currently being administered intravenously. This is due to the early stage of these therapies and the barriers that still remain for real targeted therapies such as (1) controlling lung clearance, (2) achieving a homogeneous biodistribution, (3) obtaining stable miRNA carriers for nebulization, and (4) obtaining selective delivery systems to the target cells. In addition, what is not yet known is whether the decrease in PH pathology is temporary or lasting [43]. Future advances in other miRNA therapies such as cancer are likely to help address these challenges for PH [44].

In the field of non-coding RNA therapies, combination therapies are expected to be more efficient than single-target RNA; Sindi et al. have obtained outstanding results in this direction already in PH [19]. These authors first focused their work on deciphering whether the Krüppel-like factor 2 (KLF2) signaling pathway in PAECs could be a novel molecular target to combat PH. They then demonstrated that the combination therapy of exosomal miR-181a-5p and miR-324-5p was helpful in reducing right ventricle pressure and hypertrophy, cell proliferation and angiogenesis in murine models. Interestingly, they compared single miRNA delivery with combination therapy displaying a higher efficiency for the latter. This study, in addition shows the potential use of exosomes as efficient carriers for miRNA-based therapies, illustrates perfectly how important is to detect many different miRNAs to find the best therapeutic outcome.

**Figure 5:**
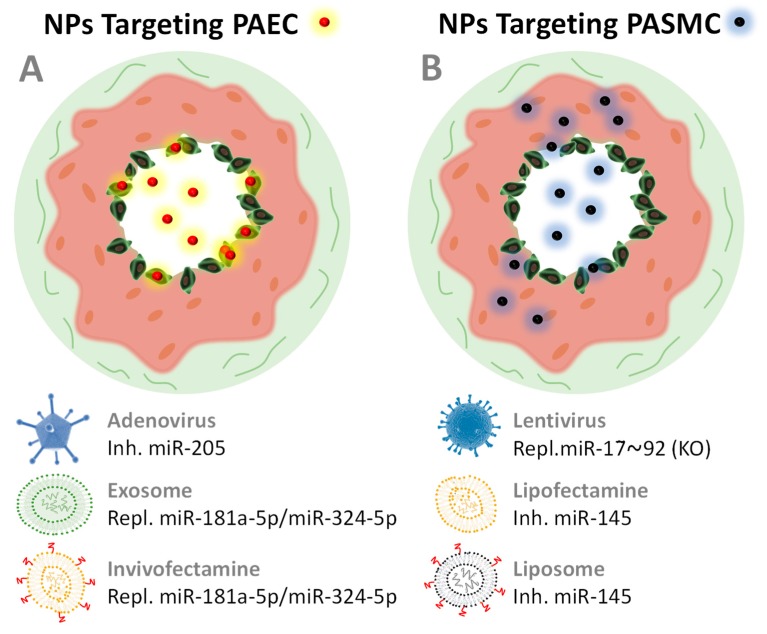
Schematic drawing of a cross section of two pulmonary arteries that develop PH and are being treated with nanopharmaceuticals (represented as small red and black spheres) that target the endothelial cells of the PAEC arteries (**A**) or smooth muscle cells PASMC (**B**). Endothelial dysfunction leads to a patent state in which NPs can leak into the pulmonary vasculature and reach PASMC more easily than in healthy arteries. The nanovectors collected in Table 3 are shown here.

Figure 5 depicts the few but promising nanovectors applied to date to treat PH with miRNA therapies and their reported cell targets (also reported in Table 3). Nanovectors may target PASMCs because of dysfunctional endothelium patency in HP [131]. However, the use of nanovectors functionalized with targeting molecules could show significant improvements to avoid side and off-target effects while improving therapeutic outcomes. Peptides and antibodies have been already applied successfully in miRNA-based therapies when targeting other organs such as heart or liver [70,132,133,134], so we are convinced that this strategy will be promptly applied in PH treatments. There is plenty of room for improvement in miRNA therapeutics for PH.

## 9. Conclusions

Although nowadays the majority of miRNA-based therapeutics in clinical trials and patents are related with cancer [43], there is a great promise for the treatment of pulmonary diseases and other pathologies. In particular pulmonary hypertension can greatly benefit from all the research that has been already advanced for miRNA-based cancer treatments. PH and cancer hold many similarities such as cell hyperproliferation and the Warburg effect that make easier to find appropriated molecular targets. We have shown that there is a long list of possible miRNA targets for developing miRNA-based treatments for PH but few studies that have already assayed their efficacy to reverse the severity of this fatal disease. However, they have shown that it is possible to reduce uncontrolled cell proliferation, reduce vascular remodeling and angiogenesis in preclinical trials. This successful early phase research reflects the need of developing robust delivery technologies. Bare miRNAs cell transfection is a very inefficient process that has been ameliorated with chemical modifications of such oligonucleotides. Unlikely, these modifications often decrease their specificity and there is a general consensus about the future role of nanovectors in the advance of the field. Until now, there are no perfect nanovectors for miRNA delivery, but we are convinced that the way to advance this type of therapy will go through the improvement of known nanotherapies, either with increased transfection efficiency, moieties to target cells, or combination therapies. All this will be aimed at improving efficacy and specificity which are currently the main goals for miRNA-loaded nanovectors applied to PH.

## Figures and Tables

**Table 1:** Examples of miRNA-based therapies applied in lung diseases.

Targeted miRNA	NV ^1^	Admin. ^2^	Type of Therapy	Disease	Effect	Application	Ref. ^3^
miR-29	None	i.v.i. ^4^	Repl. ^5^	PF ^6^	↓Col1a1 ^7^, Col3a1 ^8^ expression	BMM ^9^	[96]
miR-200c	None	int.ins. ^10^	Repl.	PF	↓ Fibroblast fibrogenic phenotype	BMM Human cells	[97]
miR-155-5p	None	intranasal	Inh. ^11^	Asthma	↓ Disease phenotype	Allergic airway disease MM ^12^	[98]
miR-101	Adenovirus	inh.	Repl.	PF	↓ Fibroblast proliferation	BMM	[99]
miR-34a	Lipid NP ^13^	s.c. ^14^	Repl.	Lung cancer	↓ Tumor growth	Lung cancer xenograph	[87]
miR-34a	Liposome	i.v.i.	Repl.	Solid tumor	↑ Antitumor activity	Humans, Phase I	[76]

^1^ Nanovector. ^2^ Administration. ^3^ Reference. ^4^ Intravenous injection. ^5^ Replacement therapy. ^6^ Pulmonary fibrosis. ^7^ Collagen1a1. ^8^ Collagen3a1. ^9^ Bleomycin murine model. ^10^ Intratracheal instillation. ^11^ Inhibition therapy. ^12^ Murine model. ^13^ Nanoparticle. ^14^ Subcutaneous injection. ↑ means increase and ↓ means decrease.

**Table 2:** Reported dysregulated miRNA biomarkers in pulmonary hypertension, origin of the obtained data, reported targets and miR functions.

miRNA	Model	Target Gene	Exp. ^1^	Function	Ref. ^2^
miR-9-1 + miR-9-3	PASMCs ^3^ from HMM ^4^	HIF-1 α	↑	Phenotypic switch	[111]
miR-17-5p + miR-20a	Bioinformatics +HEK293 cells	BMPR2	↑	Differentiation, proliferation and fibrous matrix production of PAECS ^5^ and PASMCs	[112]
miR-100	HMM, MCT ^6^	mTOR	↓	Proliferation of PASMCs	[113]
miR-124	HPAECs ^7^	PTPB1 + PKM1/M2	↓	Proliferation of PASMCs and PAFs ^8^	[114]
miR-140-5p	Bioinformatics (miRBase database)	23 genes & 7 signaling pathways	↓	Proliferation and pro-differentiation of PAECS, PASMCs & PAFs	[115]
miR-145	HMM, HPASMC	BMPR2	↑	Proliferation of PASMCs	[116]
miR-199a-5p	HPASMCs, HPAECs & HMM	SMAD3	↑	Inhibit the level of NO and promote the concentration of Ca^2+^	[117]
miR-204	PAECs	TGFBR2, α-SMA, SMAD2/7	↓	Proliferation and migration of PAECs	[118]
miR-205-5p	Hypoxic PASMCs & HMM	MICAL2 ERK1/2 signaling	↓	Proliferation of PASMCs	[110]
miR-206	PASMCs & HMM	HIF-1 α /FHL-1	↓	Promotion of cell entry into the S phase and PASMC proliferation	[119]
miR-214	Hypoxic HPASMCs, Sugen/HMM	CCNL2, LMOD1, MEF2C, PTEN	↑	Proliferation of PASMCs by suppressing cell apoptosis	[120]
miR-339	MCT murine model	FGF	↓	Proliferation of PASMC	[121]
miR-449a-5p	PASMCs from MCT	MYC	↓	Mitochondrial dysfunction and proliferation of PASMCs	[122]
miR-1281	Hypoxic HPASMCs & MCT	HDAC4	↓	Cell proliferation and migration	[123]
miR-637	HPASMCs	CDK6	↓	Increase PASMCs viability	[124]
miR-4632	HPASMCs	JUN	↓	Inhibit proliferation and promote HPASMCs apoptosis	[125]

^1^ Expression of miRNA. ^2^ Reference. ^3^ Pulmonary artery smooth muscle cells. ^4^ Hypoxic murine model. ^5^ Pulmonary artery endothelial cells. ^6^ Monocrotaline murine model. ^7^ Human pulmonary artery endothelial cells. ^8^ Pulmonary artery fibroblasts. ↑ means increase and ↓ means decrease.

**Table 3:** Relevant information about miRNA-based therapies in pulmonary hypertension.

Targeted miRNA	NV ^1^	Adm. ^2^	Therapy	Effect	Application	Target cell	Ref. ^3^
miR-17	None (Antagomir)	i.v.i. ^4^	Inh. ^5^	↓Arteriopathy ↓RVP ^6^ ↓RVH ^7^ ↑Artery acceleration time	MCT ^8^ HMM ^9^	PASMC	[128]
miR-21	None (Antagomirs)	i.v.i	Inh.	↓Arteriopathy ↓RVP	HMM	PASMC	[128]
miR-92a	None (Antagomirs)	i.v.i	Inh.	↓Arteriopathy	HMM	PASMC	[128]
miR-17~92	Lentivirus (+ miR mimics)	i.v.i.	Repl.	Restore hypoxia phenotype	PASMCs HM-KO-M ^10^	PASMC	[129]
miR-145	Lipofectamine	s.c.i. ^11^	Inh.	↓RVP ↓ Arteriopathy	HPASMC HMM	PASMC	[116]
miR-145	Liposomes ^12^	i.v.i.	Inh.	↓RVP ↓Artery thickness	Sugen/HMM	PASMC	[7]
miR-181a-5p/miR-324-5p	EV and Invivofectamine	i.v.i	Repl. ^13^	↓RVP, ↓RVH ↓Cell proliferation ↓Angiogenesis	HPAECs Sugen/HMM	PAEC	[19]
miR-204a	Invivofectamine	int.neb. ^14^	Repl.	↓Cell proliferation ↓Vascular remodeling ↓PA blood pressure	PASMCs MCT	PASMC	[109]
miR-205	Lipofectamine	in vitro	Repl.	↓PASMC proliferation	PASMC HPASMC	PASMC	[110]
miR-495	Adeno-associated virus	i.v.i.	Inh.	↓Vascular remodeling ↓Angiogenesis	Sugen/HMM	PAEC	[130]

^1^ Nanovector. ^2^ Administration. ^3^ Reference. ^4^ Intravenous injection. ^5^ Inhibition therapy. ^6^ Right Ventricle Preassure. ^7^ Right Ventricle Hypertrophy. ^8^ Monocrotaline murine model. ^9^ Hypoxic murine model. ^10^ Hypoxic murine knockout model. ^11^ Subcutaneous injection. ^12^ Nanoparticles. ^13^ Replacement therapy. ^14^ Intratracheal nebulization. ↑ means increase and ↓ means decrease.

## Abbreviations

miRNA	microRNA
siRNA	small interfering RNA
mRNA	messenger RNA
LNA	locked nucleic acid
AGO	Argonaute protein
RISC	RNA-induced silencing complex
NV	nanovector
NP	nanoparticle
PEG	polyethylene glycol
PLGA	poly(lactic-co-glycolic acid)
EV	extracellular vesicles
FDA	Food and drug administration of Unites States
EMA	European medicine agency
PA	pulmonary artery
PH	pulmonary hypertension
PAH	pulmonary artery hypertension
UTR	untranslated region
PAEC	pulmonary artery endothelial cell
HPAEC	human pulmonary artery endothelial cell
PASMC	pulmonary artery smooth muscle cell
HPASMC	human pulmonary artery smooth muscle cell
PAF	pulmonary artery fibroblast
HIF-1 α	hypoxia inducible factor 1-alpha
BMPR2	bone morphogenetic protein receptor type II
mTOR	mammalian target of rapamycin
PTPB1	polypyrimidine tract-binding protein 1
PKM1/M2	pyruvate kinase isozymes M1/M2
SMAD3	mothers against decapentaplegic homolog 3
TGFBR2	transforming growth factor beta receptor 2
α-SMA	Alpha-smooth muscle actin
SMAD2/7	mothers against decapentaplegic homolog 2/7
MICAL2	microtubule associated monooxygenase, calponin and LIM domain containing 2
ERK1/2	extracellular signal-regulated kinase ½
FHL-1	four and a half LIM domain protein
CCNL2	cyclin-L2
LMOD1	leiomodin 1
MEF2C	myocyte-specific enhancer factor 2C
PTEN	phosphatase and tensin homolog
FGF	fibroblast growth factor
MYC	master regulator of cell entry and proliferative metabolism
HDAC4	histone deacetylase 4
CDK6	cell division protein kinase 6

## References

[1] Lee R.C., Feinbaum R.L., Ambros V. (1993). The C. elegans heterochronic gene lin-4 encodes small RNAs with antisense complementarity to lin-14. Cell.

[2] Reinhart B.J., Slack F.J., Basson M., Pasquinelli A.E., Bettinger J.C., Rougvie A.E., Horvitz H.R., Ruvkun G. (2000). The 21-nucleotide let-7 RNA regulates developmental timing in Caenorhabditis elegans. Nature.

[3] Hammond S.M. (2015). An overview of microRNAs. Adv. Drug Deliv. Rev..

[4] Djebali S., Davis C.A., Merkel A., Dobin A., Lassmann T., Mortazavi A.M., Tanzer A., Lagarde J., Lin W., Schlesinger F. (2012). Landscape of transcription in human cells. Nature.

[5] Lam J.K.-W., Chow M.Y.T., Zhang Y., Leung S.W.S. (2015). siRNA Versus miRNA as Therapeutics for Gene Silencing. Mol. Ther. Nucleic Acids.

[6] Bartoszewski R., Sikorski A. (2019). Editorial focus: Understanding off-target effects as the key to successful RNAi therapy. Cell. Mol. Boil. Lett..

[7] McLendon J., Joshi S.R., Sparks J., Matar M., Fewell J.G., Abe K., Oka M., McMurtry I.F., Gerthoffer W. (2015). Lipid nanoparticle delivery of a microRNA-145 inhibitor improves experimental pulmonary hypertension. J. Control. Release.

[8] Van Zandwijk N., Pavlakis N., Kao S., Linton A., Boyer M., Clarke S., Huynh Y., Chrzanowska A., Fulham M., Bailey D.L. (2017). Safety and activity of microRNA-loaded minicells in patients with recurrent malignant pleural mesothelioma: A first-in-man, phase 1, open-label, dose-escalation study. Lancet Oncol..

[9] Lindow M., Kauppinen S. (2012). Discovering the first microRNA-targeted drug. J. Cell Boil..

[10] Fujita K., Mimura S., Iwama H., Nakahara M., Oura K., Tadokoro T., Nomura T., Tani J., Yoneyama H., Morishita A. (2018). Serum miRNAs Predicting Sustained HBs Antigen Reduction 48 Weeks after Pegylated Interferon Therapy in HBe Antigen-Negative Patients. Int. J. Mol. Sci..

[11] Haas G., Cetin S., Messmer M., Chane-Woon-Ming B., Terenzi O., Chicher J., Kuhn L., Hammann P., Pfeffer S. (2016). Identification of factors involved in target RNA-directed microRNA degradation. Nucleic Acids Res..

[12] A Lennox K., Behlke M.A. (2011). Chemical modification and design of anti-miRNA oligonucleotides. Gene Ther..

[13] Duygu B., Juni R., Ottaviani L., Bitsch N., Wit J.B., De Windt L.J., Martins P.A.D.C. (2019). Comparison of different chemically modified inhibitors of miR-199b in vivo. Biochem. Pharmacol..

[14] Wu H., Fan H., Shou Z., Xu M., Chen Q., Ai C., Dong Y., Liu Y., Nan Z., Wang Y. (2019). Extracellular vesicles containing miR-146a attenuate experimental colitis by targeting TRAF6 and IRAK1. Int. Immunopharmacol..

[15] Thomas M., Lange-Grünweller K., Dayyoub E., Bakowsky U., Weirauch U., Aigner A., Hartmann R.K., Grünweller A. (2012). PEI-complexed LNA antiseeds as miRNA inhibitors. RNA Boil..

[16] Naseri Z., Oskuee R.K., Jaafari M.R., Moghadam M.F. (2018). Exosome-mediated delivery of functionally active miRNA-142-3p inhibitor reduces tumorigenicity of breast cancer in vitro and in vivo. Int. J. Nanomed..

[17] Cool C.D., Kuebler W.M., Bogaard H.J., Spiekerkoetter E., Nicolls M.R., Voelkel N.F. (2020). The Hallmarks of Severe Pulmonary Arterial Hypertension: The Cancer Hypothesis - Ten years later. Am. J. Physiol. Cell. Mol. Physiol..

[18] Sommer N., Ghofrani A., Pak O., Bonnet S., Provencher S., Sitbon O., Rosenkranz S., Hoeper M.M., Kiely D.G. (2020). Current and future treatments of pulmonary arterial hypertension. Br. J. Pharmacol..

[19] Sindi H.A., Russomanno G., Satta S., Abdul-Salam V.B., Jo K.B., Qazi-Chaudhry B., Ainscough A.J., Szulcek R., Bogaard H.J., Morgan C.C. (2020). Therapeutic potential of KLF2-induced exosomal microRNAs in pulmonary hypertension. Nat. Commun..

[20] Kim Y.K., Kim B., Kim V.N. (2016). Re-evaluation of the roles of DROSHA, Export in 5, and DICER in microRNA biogénesis. Proc. Natl. Acad. Sci. USA.

[21] Kim Y., Kim V.N. (2012). MicroRNA factory: RISC assembly from precursor microRNAs. Mol. Cell.

[22] Treiber T., Treiber N., Meister G. (2019). Publisher Correction: Regulation of microRNA biogenesis and its crosstalk with other cellular pathways. Nat. Rev. Mol. Cell Boil..

[23] Michlewski G., Guil S., Semple C.A., Caceres J.F. (2008). Posttranscriptional Regulation of miRNAs Harboring Conserved Terminal Loops. Mol. Cell.

[24] Michlewski G., Caceres J.F. (2010). Antagonistic role of hnRNP A1 and KSRP in the regulation of let-7a biogenesis. Nat. Struct. Mol. Boil..

[25] Yi R., Qin Y., Macara I.G., Cullen B.R. (2003). Exportin-5 mediates the nuclear export of pre-microRNAs and short hairpin RNAs. Genome Res..

[26] Gerlach C.V., Vaidya V.S. (2017). MicroRNAs in injury and repair. Arch. Toxicol..

[27] McDermott A.M., Heneghan H., Miller N., Kerin M. (2011). The Therapeutic Potential of MicroRNAs: Disease Modulators and Drug Targets. Pharm. Res..

[28] Wu L., Zhou H., Zhang Q., Zhang J., Ni F., Liu C., Qi Y. (2010). DNA Methylation Mediated by a MicroRNA Pathway. Mol. Cell.

[29] Khraiwesh B., Arif M.A., Seumel G.I., Ossowski S., Weigel D., Reski R., Frank W. (2010). Transcriptional Control of Gene Expression by MicroRNAs. Cell.

[30] Agostini M., Ganini C., Candi E., Melino G. (2020). The role of noncoding RNAs in epithelial cancer. Cell Death Discov..

[31] Morozova N., Zinovyev A., Nonne N., Pritchard L.-L., Gorban A., Harel-Bellan A. (2012). Kinetic signatures of microRNA modes of action. RNA.

[32] Zinovyev A., Morozova N., Gorban A., Harel-Belan A. (2012). Mathematical Modeling of microRNA–Mediated Mechanisms of Translation Repression. Results Probl. Cell Differ..

[33] Lai X., Bhattacharya A., Schmitz U., Kunz M., Vera J., Wolkenhauer O. (2013). A Systems’ Biology Approach to Study MicroRNA-Mediated Gene Regulatory Networks. BioMed. Res. Int..

[34] miRBase: The MicroRNA Database. http://www.mirbase.org.

[35] Masson S., Batkai S., Beermann J., Bär C., Pfanne A., Thum S., Magnoli M., Balconi G., Nicolosi G.L., Tavazzi L. (2017). Circulating microRNA-132 levels improve risk prediction for heart failure hospitalization in patients with chronic heart failure. Eur. J. Hear. Fail..

[36] Canivet C., Napoléon B., Palazzo L., Flori N., Guibert P., Piessen G., Farges-Bancel D., Seitz J.-F. (2018). A prospective clinical and biological database for pancreatic adenocarcinoma: The BACAP cohort. BMC Cancer.

[37] Ma L., Zheng X., Yang Y., Wang J., Xu Y., Wang B. (2018). Epigenetic differences of chronic hepatitis B in different TCM syndromes. Medicine.

[38] Kho A.T., McGeachie M., Moore K.G., Sylvia J.M., Weiss S.T., Tantisira K. (2018). Circulating microRNAs and prediction of asthma exacerbation in childhood asthma. Respir. Res..

[39] Romeo P., Colombo C., Granata R., Calareso G., Gualeni A.V., Dugo M., De Cecco L., Rizzetti M.G., Zanframundo A., Aiello A. (2018). Circulating miR-375 as a novel prognostic marker for metastatic medullary thyroid cancer patients. Endocr. Relat. Cancer.

[40] Fan B., Shen C., Wu M., Zhao J., Guo Q., Luo Y. (2018). miR-17-92 cluster is connected with disease progression and oxaliplatin/capecitabine chemotherapy efficacy in advanced gastric cancer patients: A preliminary study. Medicine (Baltimore).

[41] Fernández-Ruiz J.C., Ramos-Remus C., Corona J.S., Castillo-Ortiz J.D., Castañeda-Sánchez J.J., Bastian Y., Romo-García M.F., Ochoa-González F., Monsivais-Urenda A.E., González-Amaro R. (2018). Analysis of miRNA expression in patients with rheumatoid arthritis during remission and relapse after a 5-year trial of tofacitinib treatment. Int. Immunopharmacol..

[42] Krintel S.B., Dehlendorff C., Hetland M.L., Hørslev-Petersen K., Andersen K.K., Junker P., Pødenphant J., Ellingsen T., Ahlquist P., Lindegaard H.M. (2015). Prediction of treatment response to adalimumab: A double-blind placebo-controlled study of circulating microRNA in patients with early rheumatoid arthritis. Pharmacogenomics J..

[43] Chakraborty C., Sharma A.R., Sharma G., Doss C.G.P., Lee S.-S. (2017). Therapeutic miRNA and siRNA: Moving from Bench to Clinic as Next Generation Medicine. Mol. Ther. Nucleic Acids.

[44] Yin W., Rogge M. (2019). TargetingRNA: A Transformative Therapeutic Strategy. Clin. Transl. Sci..

[45] Lieberman J. (2018). Tapping the RNA world for therapeutics. Nat. Struct. Mol. Boil..

[46] Shen X., Corey D.R. (2018). Chemistry, mechanism and clinical status of antisense oligonucleotides and duplex RNAs. Nucleic Acids Res..

[47] Yu A., Jian C., Yu A.H., Tu M.-J. (2019). RNA therapy: Are we using the right molecules?. Pharmacol. Ther..

[48] Scherr M., Eder M. (2007). Gene Silencing by Small Regulatory RNAs in Mammalian Cells. Cell Cycle.

[49] Obika S., Nanbu D., Hari Y., Morio K.-I., In Y., Ishida T., Imanishi T. (1997). Synthesis of 2′-O,4′-C-methyleneuridine and -cytidine. Novel bicyclic nucleosides having a fixed C3, -endo sugar puckering. Tetrahedron Lett..

[50] Kumar R., Singh S.K., Koshkin A.A., Rajwanshi V.K., Meldgaard M., Wengel J. (1998). The first analogues of LNA (locked nucleic acids): Phosphorothioate-LNA and 2’-thio-LNA. Bioorganic Med. Chem. Lett..

[51] Krützfeldt J., Rajewsky N., Braich R., Rajeev K.G., Tuschl T., Manoharan M., Stoffel M. (2005). Silencing of microRNAs in vivo with ‘antagomirs’. Nature.

[52] Wang Z. (2010). The Guideline of the Design and Validation of MiRNA Mimics. Adv. Struct. Saf. Stud..

[53] Reid G., Kao S.C., Pavlakis N., Brahmbhatt H., MacDiarmid J., Clarke S., Boyer M., Van Zandwijk N. (2016). Clinical development of TargomiRs, a miRNA mimic-based treatment for patients with recurrent thoracic cancer. Epigenomics.

[54] Lanford R.E., Hildebrandt-Eriksen E.S., Petri A., Persson R., Lindow M., Munk M.E., Kauppinen S., Ørum H. (2009). Therapeutic Silencing of MicroRNA-122 in Primates with Chronic Hepatitis C Virus Infection. Science.

[55] Wang Y., Sheng G., Juranek S., Tuschl T., Patel D.J. (2008). Structure of the guide-strand-containing argonaute silencing complex. Nature.

[56] Schmidt M.F., Korb O., Abell C. (2013). MicroRNA-Specific Argonaute 2 Protein Inhibitors. ACS Chem. Boil..

[57] Fujita Y., Takeshita F., Mizutani T., Ohgi T., Kuwano K., Ochiya T. (2013). A novel platform to enable inhaled naked RNAi medicine for lung cancer. Sci. Rep..

[58] Schlosser K., Taha M., Stewart D.J. (2018). Systematic Assessment of Strategies for Lung-targeted Delivery of MicroRNA Mimics. Theranostics.

[59] Sun X., Guo Q., Wei W.-H., Robertson S., Yuan Y., Luo X. (2019). Current Progress on MicroRNA-Based Gene Delivery in the Treatment of Osteoporosis and Osteoporotic Fracture. Int. J. Endocrinol..

[60] Segura-Ibarra V., Wu S., Hassan N., Moran-Guerrero J.A., Ferrari M., Guha A., Karmouty-Quintana H., Blanco E. (2018). Nanotherapeutics for Treatment of Pulmonary Arterial Hypertension. Front. Physiol..

[61] Yang J., Fan Z., Yang J., Ding J., Yang C., Chen L. (2016). MicroRNA-24 Attenuates Neointimal Hyperplasia in the Diabetic Rat Carotid Artery Injury Model by Inhibiting Wnt4 Signaling Pathway. Int. J. Mol. Sci..

[62] Lee H.K., Finniss S., Cazacu S., Xiang C., Brodie C. (2014). Mesenchymal Stem Cells Deliver Exogenous miRNAs to Neural Cells and Induce Their Differentiation and Glutamate Transporter Expression. Stem Cells Dev..

[63] Zeng Y., Wagner E.J., Cullen B. (2002). Both Natural and Designed Micro RNAs Can Inhibit the Expression of Cognate mRNAs When Expressed in Human Cells. Mol. Cell.

[64] Stegmeier F., Hu G., Rickles R.J., Hannon G.J., Elledge S.J. (2005). A lentiviral microRNA-based system for single-copy polymerase II-regulated RNA interference in mammalian cells. Proc. Natl. Acad. Sci. USA.

[65] Couto L.B., High K. (2010). Viral vector-mediated RNA interference. Curr. Opin. Pharmacol..

[66] Ingato D., Lee J.U., Sim S.J., Kwon Y.J. (2016). Good things come in small packages: Overcoming challenges to harness extracellular vesicles for therapeutic delivery. J. Control. Release.

[67] Sancho-Albero M., Rubio-Ruiz B., Pérez-López A.M., Sebastian V., Martin-Duque P., Arruebo M., María S.-A., Unciti-Broceta A. (2019). Cancer-derived exosomes loaded with ultrathin palladium nanosheets for targeted bioorthogonal catalysis. Nat. Catal..

[68] Momen-Heravi F., Bala S., Bukong T., Szabo G. (2014). Exosome-mediated delivery of functionally active miRNA-155 inhibitor to macrophages. Nanomed. Nanotechnol. Boil. Med..

[69] Fuster-Matanzo A., Gessler F., Leonardi T., Iraci N., Pluchino S. (2015). Acellular approaches for regenerative medicine: On the verge of clinical trials with extracellular membrane vesicles?. Stem Cell Res. Ther..

[70] Bai Z., Wei J., Yu C., Han X., Qin X., Zhang C., Zhang T.-T., Li L., Huang W. (2019). Non-viral nanocarriers for intracellular delivery of microRNA therapeutics. J. Mater. Chem. B.

[71] Yang C., Yin M., Xu G., Lin W.-J., Chen J., Zhang Y., Feng T., Huang P., Chen C.-K., Yong K.-T. (2019). Biodegradable Polymers as a Noncoding miRNA Nanocarrier for Multiple Targeting Therapy of Human Hepatocellular Carcinoma. Adv. Heal. Mater..

[72] Byk T., Haddada H., Vainchenker W., Louache F. (1998). Lipofectamine and related cationic lipids strongly improve adenoviral infection efficiency of primitive human hematopoietic cells. Hum. Gene Ther..

[73] Lin C.-W., Jan M.-S., Kuo J.-H.S. (2017). The vector-related influences of autophagic microRNA delivery by Lipofectamine 2000 and polyethylenimine 25K on mouse embryonic fibroblast cells. Eur. J. Pharm. Sci..

[74] Cardarelli F., Digiacomo L., Marchini C., Amici A., Salomone F., Fiume G., Rossetta A., Gratton E., Pozzi D., Caracciolo G. (2016). The intracellular trafficking mechanism of Lipofectamine-based transfection reagents and its implication for gene delivery. Sci. Rep..

[75] Felgner P.L., Gadek T.R., Holm M., Roman R., Chan H.W., Wenz M., Northrop J.P., Ringold G.M., Danielsen M. (1987). Lipofection: A highly efficient, lipid-mediated DNA-transfection procedure. Proc. Natl. Acad. Sci. USA.

[76] Beg M.S., Brenner A.J., Sachdev J., Borad M., Kang Y.-K., Stoudemire J., Smith S., Bader A.G., Kim S., Hong D.S. (2016). Phase I study of MRX34, a liposomal miR-34a mimic, administered twice weekly in patients with advanced solid tumors. Investig. New Drugs.

[77] Rietwyk S., Peer D. (2017). Next-Generation Lipids in RNA Interference Therapeutics. ACS Nano.

[78] Escudero A., Carregal-Romero S., Miguel-Coello A.B., Ruíz-Cabello J. (2020). Engineered polymeric nanovehicles for drug delivery. Cluster Beam Deposition of Functional Nanomaterials and Devices.

[79] Di Silvio D., Martínez-Moro M., Salvador C., de los Angeles Ramirez M., Caceres-Velez P.R., Ortore M.G., Dupin D., Andreozzi P., Moya S.E. (2019). Self-assembly of poly(allylamine)/siRNA nanoparticles, their intracellular fate and siRNA delivery. J. Colloid Interface Sci..

[80] Dzmitruk V., Apartsin E., Ihnatsyeu-Kachan A., Abashkin V., Shcharbin D., Bryszewska M. (2018). Dendrimers Show Promise for siRNA and microRNA Therapeutics. Pharmaceutical.

[81] Froehlich E., Mandeville J.-S., Kreplak L., Tajmir-Riahi H.-A. (2011). Aggregation and Particle Formation of tRNA by Dendrimers. Biomacromolecules.

[82] Devalliere J., Chang W.G., Andrejecsk J.W., Abrahimi P., Cheng C.J., Jane-Wit D., Saltzman W.M., Pober J.S. (2013). Sustained delivery of proangiogenic microRNA-132 by nanoparticle transfection improves endothelial cell transplantation. FASEB J..

[83] Liang G., Zhu Y., Jing A., Wang J., Hu F., Feng W., Xiao Z., Chen B. (2016). Cationic microRNA-delivering nanocarriers for efficient treatment of colon carcinoma in xenograft model. Gene Ther..

[84] De Backer L., Braeckmans K., Stuart M.C.A., Demeester J., De Smedt S.C., Raemdonck K. (2015). Bio-inspired pulmonary surfactant-modified nanogels: A promising siRNA delivery system. J. Control. Release.

[85] Strozyk M.S., Carregal-Romero S., Henriksen-Lacey M., Brust M., Liz-Marzán L.M. (2017). Biocompatible, Multiresponsive Nanogel Composites for Codelivery of Antiangiogenic and Chemotherapeutic Agents. Chem. Mater..

[86] Li Y., Duo Y., Bi J., Zeng X., Mei L., Bao S., He L., Shan A., Zhang Y., Yu X. (2018). Targeted delivery of anti-miR-155 by functionalized mesoporous silica nanoparticles for colorectal cancer therapy. Int. J. Nanomed..

[87] Ott A., Yu X., Hartmann R., Rejman J., Schütz A., Ochs M., Parak W.J., Carregal-Romero S. (2015). Light-Addressable and Degradable Silica Capsules for Delivery of Molecular Cargo to the Cytosol of Cells. Chem. Mater..

[88] Di Mauro V., Iafisco M., Salvarani N., Vacchiano M., Carullo P., Ramírez-Rodríguez G.B., Patrício T., Tampieri A., Miragoli M., Catalucci D. (2016). Bioinspired negatively charged calcium phosphate nanocarriers for cardiac delivery of MicroRNAs. Nanomed.

[89] Sokolova V., Rotan O., Klesing J., Nalbant P., Buer J., Knuschke T., Westendorf A.M., Epple M. (2012). Calcium phosphate nanoparticles as versatile carrier for small and large molecules across cell membranes. J. Nanoparticle Res..

[90] Wu J., Huang J., Kuang S., Chen J., Li X., Chen B., Wang J., Cheng D., Shuai X. (2019). Synergistic MicroRNA Therapy in Liver Fibrotic Rat Using MRI-Visible Nanocarrier Targeting Hepatic Stellate Cells. Adv. Sci..

[91] Osorio-Querejeta I., Carregal-Romero S., Ayerdi-Izquierdo A., Mäger I., Nash L.A., Wood M., Egimendia A., Betanzos M., Alberro A., Iparraguirre L. (2020). MiR-219a-5p Enriched Extracellular Vesicles Induce OPC Differentiation and EAE Improvement More Efficiently Than Liposomes and Polymeric Nanoparticles. Pharmaceutical.

[92] Maltby S., Plank M., Tay H.L., Collison A., Foster P.S. (2016). Targeting MicroRNA Function in Respiratory Diseases: Mini-Review. Front. Physiol..

[93] Guo Z.M., He B., Jin H.W., Zhang H.R., Dai W.B., Zhang L.R., Zhang H., Wang X.Q., Wang J.C., Zhang X. (2014). Targeting efficiency of RGD-modified nanocarriers with different ligand intervals in response to integrin alpha v beta 3 clustering. Biomaterials.

[94] Stolzenburg L., Harris A. (2018). The role of microRNAs in chronic respiratory disease: Recent insights. Boil. Chem..

[95] Bonnet S., Boucherat O., Paulin R., Wu D., Hindmarch C.C.T., Archer S.L., Song R., Moore J.B., Provencher S., Zhang L. (2020). Clinical value of non-coding RNAs in cardiovascular, pulmonary, and muscle diseases. Am. J. Physiol. Physiol..

[96] Montgomery R.L., Yu G., A Latimer P., Stack C., Robinson K., Dalby C.M., Kaminski N., Rooij E. (2014). Micro RNA mimicry blocks pulmonary fibrosis. EMBO Mol. Med..

[97] Yang S., Banerjee S., Freitas A., Sanders Y.Y., Ding Q., Matalon S., Thannickal V.J., Abraham E., Liu G. (2012). Participation of miR-200 in Pulmonary Fibrosis. Am. J. Pathol..

[98] Plank M.W., Maltby S., Tay H.L., Stewart J., Eyers F., Hansbro P.M., Foster P.S. (2015). MicroRNA Expression Is Altered in an Ovalbumin-Induced Asthma Model and Targeting miR-155 with Antagomirs Reveals Cellular Specificity. PLoS ONE.

[99] Huang C., Xiao X., Yang Y., Mishra A., Liang Y., Zeng X., Yang X., Xu D., Blackburn M.R., Henke C.A. (2017). MicroRNA-101 attenuates pulmonary fibrosis by inhibiting fibroblast proliferation and activation. J. Boil. Chem..

[100] Archer S.L., Weir E.K., Wilkins M.R. (2010). The Basic Science of Pulmonary Arterial Hypertension for Clinicians New concepts and experimental therapies. Circulation.

[101] Budhiraja R., Tuder R.M., Hassoun P.M. (2004). Endothelial Dysfunction in Pulmonary Hypertension. Circulation.

[102] Izquierdo-Garcia J.L., Arias T., Rojas Y., Garcia-Ruiz V., Santos A., Martin-Puig S., Ruíz-Cabello J. (2018). Metabolic Reprogramming in the Heart and Lung in a Murine Model of Pulmonary Arterial Hypertension. Front. Cardiovasc. Med..

[103] Izquierdo-García J.L., Fadon L., Beraza M., Cossio U., Llop J., Ruiz-Cabello J. (2019). In-vivo lung molecular imaging of choline metabolism in a rat model of pulmonary arterial hypertension. Pulm. Hypertens..

[104] Ranchoux B., Harvey L., Ayon R.J., Babicheva A., Bonnet S., Chan S.Y., Yuan J.X.-J., Perez V.A.D.J. (2017). Endothelial dysfunction in pulmonary arterial hypertension: An evolving landscape (2017 Grover Conference Series). Pulm. Circ..

[105] Ranchoux B., Antigny F., Rucker-Martin C., Hautefort A., Péchoux C., Bogaard H.J., Dorfmüller P., Remy S., Lecerf F., Planté S. (2015). Endothelial-to-Mesenchymal Transition in Pulmonary Hypertension. Circulation.

[106] Humbert M., Ghofrani H.A. (2015). The molecular targets of approved treatments for pulmonary arterial hypertension. Thorax.

[107] Chun H.J., Bonnet S., Chan S.Y. (2017). Translational Advances in the Field of Pulmonary Hypertension.Translating MicroRNA Biology in Pulmonary Hypertension. It Will Take More Than “miR” Words. Am. J. Respir. Crit. Care Med..

[108] Shi Y., Huang J., Zhou J., Liu Y., Fu X., Li Y., Yin G., Wen J. (2015). MicroRNA-204 inhibits proliferation, migration, invasion and epithelial-mesenchymal transition in osteosarcoma cells via targeting Sirtuin 1. Oncol. Rep..

[109] Courboulin A., Paulin R., Giguere N.J., Saksouk N., Perreault T., Meloche J., Paquet E.R., Biardel S., Provencher S., Côté J. (2011). Role for miR-204 in human pulmonary arterial hypertension. J. Cell Boil..

[110] Tao W., Sun W., Zhu H., Zhang J. (2019). miR-205-5p suppresses pulmonary vascular smooth muscle cell proliferation by targeting MICAL2-mediated Erk1/2 signaling. Microvasc. Res..

[111] Shan F., Li J., Huang Q.-Y. (2014). HIF-1 Alpha-Induced Up-Regulation of miR-9 Contributes to Phenotypic Modulation in Pulmonary Artery Smooth Muscle Cells During Hypoxia. J. Cell. Physiol..

[112] Brock M., Trenkmann M., Gay R.E., Michel B.A., Gay S., Fischler M., Ulrich S., Speich R., Huber L.C. (2009). Interleukin-6 modulates the expression of the bone morphogenic protein receptor type II through a novel STAT3-microRNA cluster 17/92 pathway. Circ. Res..

[113] Wang A.-P., Li X.-H., Gong S.-X., Li W.-Q., Hu C.-P., Zhang Z., Li Y.-J. (2015). miR-100 suppresses mTOR signaling in hypoxia-induced pulmonary hypertension in rats. Eur. J. Pharmacol..

[114] Caruso P., Dunmore B.J., Schlosser K., Schoors S., Dos Santos C.C., Perez-Iratxeta C., Lavoie J.R., Zhang H., Long L., Flockton A.R. (2017). Identification of MicroRNA-124 as a Major Regulator of Enhanced Endothelial Cell Glycolysis in Pulmonary Arterial Hypertension via PTBP1 (Polypyrimidine Tract Binding Protein) and Pyruvate Kinase M2. Circulation.

[115] Li F., Shi W., Wan Y., Wang Q., Feng W., Yan X., Wang J., Chai L., Zhang Q., Li M. (2017). Prediction of target genes for miR-140-5p in pulmonary arterial hypertension using bioinformatics methods. FEBS Open Bio.

[116] Huber L.C., Brock M., Caruso P., Hc S., Ra M., Km M. (2012). Faculty Opinions recommendation of A role for miR-145 in pulmonary arterial hypertension: Evidence from mouse models and patient samples. Fac. Opin. Post Publ. Peer Rev. Biomed. Lit..

[117] Liu Y., Liu G., Zhang H., Wang J. (2016). MiRNA-199a-5p influences pulmonary artery hypertension via downregulating Smad3. Biochem. Biophys. Res. Commun..

[118] Yu H., Xu M., Dong Y., Liu J., Li Y., Mao W., Wang J., Wang L. (2018). 1,25(OH)2D3 attenuates pulmonary arterial hypertension via microRNA-204 mediated Tgfbr2/Smad signaling. Exp. Cell Res..

[119] Jalali S., Ramanathan G.K., Parthasarathy P.T., Aljubran S., Galam L., Yunus A., García S., Cox R.R., Lockey R.F., Kolliputi N. (2012). Mir-206 Regulates Pulmonary Artery Smooth Muscle Cell Proliferation and Differentiation. PLoS ONE.

[120] Liu H., Tao Y., Chen M., Yu J., Li W.-J., Tao L., Li Y., Li F. (2016). Upregulation of MicroRNA-214 Contributes to the Development of Vascular Remodeling in Hypoxia-induced Pulmonary Hypertension Via Targeting CCNL2. Sci. Rep..

[121] Chen J., Cui X., Li L., Qu J., Raj J.U., Gou D. (2017). MiR-339 inhibits proliferation of pulmonary artery smooth muscle cell by targeting FGF signaling. Physiol. Rep..

[122] Zhang C., Ma C., Zhang L., Zhang L., Zhang F., Ma M., Zheng X., Mao M., Shen T., Zhu D. (2019). MiR-449a-5p mediates mitochondrial dysfunction and phenotypic transition by targeting Myc in pulmonary arterial smooth muscle cells. J. Mol. Med..

[123] Li Y., Li L., Qian Z., Lin B., Chen J., Luo Y., Qu J., Raj J.U., Gou D. (2018). Phosphatidylinositol 3-Kinase–DNA Methyltransferase 1–miR-1281–Histone Deacetylase 4 Regulatory Axis Mediates Platelet-Derived Growth Factor–Induced Proliferation and Migration of Pulmonary Artery Smooth Muscle Cells. J. Am. Hear. Assoc..

[124] Sang H.-Y., Jin Y.-L., Zhang W.-Q., Chen L. (2016). Downregulation of microRNA-637 Increases Risk of Hypoxia-Induced Pulmonary Hypertension by Modulating Expression of Cyclin Dependent Kinase 6 (CDK6) in Pulmonary Smooth Muscle Cells. Med. Sci. Monit..

[125] Qian Z., Li Y., Chen J., Li X., Gou D. (2017). miR-4632 mediates PDGF-BB-induced proliferation and antiapoptosis of human pulmonary artery smooth muscle cells via targeting cJUN. Am. J. Physiol. Physiol..

[126] Santulli G. (2015). MicroRNAs and Endothelial (Dys) Function. J. Cell. Physiol..

[127] Sun C.-K., Zhen Y.-Y., Lu H.-I., Sung P.-H., Chang L.-T., Tsai T.-H., Sheu J.-J., Chen Y.-L., Chua S., Chang H.-W. (2014). Reducing TRPC1 Expression through Liposome-Mediated siRNA Delivery Markedly Attenuates Hypoxia-Induced Pulmonary Arterial Hypertension in a Murine Model. Stem Cells Int..

[128] Pullamsetti S.S., Doebele C., Fischer A., Savai R., Kojonazarov B., Dahal B.K., Ghofrani H.A., Weissmann N., Grimminger F., Bonauer A. (2012). Inhibition Of MicroRNA-17 Improves Lung And Heart Function In Experimental Pulmonary Hypertension. Am. J. Respir. Crit. Care Med..

[129] Chen T., Zhou G., Zhou Q., Tang H., Ibe J.C.F., Cheng H., Gou D., Chen J., Yuan J.X.-J., Raj J.U. (2015). Loss of MicroRNA-17∼92 in Smooth Muscle Cells Attenuates Experimental Pulmonary Hypertension via Induction of PDZ and LIM Domain 5. Am. J. Respir. Crit. Care Med..

[130] Fu J., Bai P., Chen Y., Yu T., Li F. (2019). Inhibition of miR-495 Improves Both Vascular Remodeling and Angiogenesis in Pulmonary Hypertension. J. Vasc. Res..

[131] Segura-Ibarra V., Amione-Guerra J., Cruz-Solbes A.S., Cara F.E., Iruegas-Nunez D.A., Wu S., Youker K.A., Bhimaraj A., Torre-Amione G., Ferrari M. (2017). Rapamycin nanoparticles localize in diseased lung vasculature and prevent pulmonary arterial hypertension. Int. J. Pharm..

[132] Miragoli M., Ceriotti P., Iafisco M., Vacchiano M., Salvarani N., Alogna A., Carullo P., Ramírez-Rodríguez G.B., Patrício T., Degli Esposti L. (2018). Inhalation of peptide-loaded nanoparticles improves heart failure. Sci. Transl. Med..

[133] Kulakovskii V.D., Bacher G., Weigand R., Kümmell T., Forchel A., Borovitskaya E., Leonardi K., Hommel S. (1999). Fins Structure of Biexction Emission in Symmetric and Aymmetric CdSe/ZnSe Singel Quantum Dots. Phys. Rev. Lett..

[134] Torres A.G., Fabani M.M., Vigorito E., Williams N., Al-Obaidi N., Wojciechowski F., Hudson R.H.E., Seitz O., Gait M.J. (2011). Chemical structure requirements and cellular targeting of microRNA-122 by peptide nucleic acids anti-miRs. Nucleic Acids Res..

